# Spatiotemporal analysis of the surface urban heat island (SUHI), air pollution and disease pattern: an applied study on the city of Granada (Spain)

**DOI:** 10.1007/s11356-023-26564-7

**Published:** 2023-03-27

**Authors:** David Hidalgo-García, Julián Arco-Díaz

**Affiliations:** Technical Superior School of Building Engineering, University of Granada, Fuente Nueva Campus, 18071 Granada, Spain

**Keywords:** Land surface temperature, Surface urban heat island, Urban hotspots, Sentinel 3, Environmental pollution, Diseases, Cancer

## Abstract

There is worldwide concern about how climate change —which involves rising temperatures— may increase the risk of contracting and developing diseases, reducing the quality of life. This study provides new research that takes into account parameters such as land surface temperature (LST), surface urban heat island (SUHI), urban hotspot (UHS), air pollution (SO_2_, NO_2_, CO, O_3_ and aerosols), the normalized difference vegetation index (NDVI), the normalized difference building index (NDBI) and the proportion of vegetation (PV) that allows evaluating environmental quality and establishes mitigation measures in future urban developments that could improve the quality of life of a given population. With the help of Sentinel 3 and 5P satellite images, we studied these variables in the context of Granada (Spain) during the year 2021 to assess how they may affect the risk of developing diseases (stomach, colorectal, lung, prostate and bladder cancer, dementia, cerebrovascular disease, liver disease and suicide). The results, corroborated by the statistical analysis using the Data Panel technique, indicate that the variables LST, SUHI and daytime UHS, NO_2_, SO_2_ and NDBI have important positive correlations above 99% (*p* value: 0.000) with an excess risk of developing these diseases. Hence, the importance of this study for the formulation of healthy policies in cities and future research that minimizes the excess risk of diseases.

## Introduction

In recent decades, global warming, high rates of air pollution and extreme weather events have become urgent challenges facing humanity (Kovats et al. 2005; Song et al. 2020). The modification of land cover generated by the expansion of urbanized areas, due to population growth, is a process with great repercussions in terms of climate change (Li et al. 2011). Currently, 50% of the world’s population lives in urban areas, yet according to a United Nations report, by 2050, this figure will increase to 70% (UN 2018). Thus, over the next 30 years, urban areas will have an additional 2500 million inhabitants (Mukherjee and Singh 2020), meaning an increase in urban coverage of approximately 1,527,000 km^2^ (Schneider et al. 2010), although the main driver of the expansion of transport, industry and economic growth is urbanization, altering local climates by increasing the land surface temperature (LST) (Scolozzi and Geneletti 2012; Song et al. 2020). Such development produce increases in environmental pollution in urban areas, likewise contributing to the LST or global warming. Recent investigations affirm a positive correlation between LST, pollution and changes in coverage in urban areas, with a negative correlation between LST and green areas (Hu et al. 2020; Shafizadeh-Moghadam et al. 2020; Tomlinson et al. 2011). Urban areas usually have higher temperatures than rural ones as well as higher concentrations of atmospheric pollutants. Urban green areas have lower temperatures than urban areas (Hua et al. 2020; Karakuş 2019; Tsou et al. 2017; Yang et al. 2020a). These circumstances, together with the phenomenon of urban climate change called Urban Heat Island (UHI), mean that urban areas suffer the greatest increases in temperature, its intensity being increased by multiple human activities (Santamouris 2020). This line has been corroborated by numerous studies. Thus, the studies by Tuholske et al. (2021) carried out on 13,115 urban settlements between 1983 and 2016 reported that areas with temperatures greater than 30 ºC increased by 200% between the indicated dates, affecting a total of 1.7 billion people. A recent study found that the annual average temperature of a city with a population of over one million is between 1 and 3 K higher than that of the surrounding rural areas (Khamchiangta and Dhakal 2019). There are numerous studies (Abrar et al. 2022; Liou et al. 2021; Shahfahad et al. 2022) on the evolution of temperatures and the risk of exposure to heat in various urban areas of the planet that report significant increases in temperatures which, in turn, increase the index of heat stress minimizing thermal comfort. Therefore, it is proved that the UHI is known to generate a series of environmental, climatic and socioeconomic problems that affect the quality of life of urban inhabitants (Dwivedi and Mohan 2018; Macintyre et al. 2018; Rozos et al. 2013). These problems include the reduction of biodiversity (Čeplová et al. 2017), degradation of air and water quality (Feizizadeh and Blaschke 2013), detrimental effects on the climate (Sarrat et al. 2006), changes in the energy balance (Arnfield 2003) and the increased cost of energy (Santamouris 2020). When the UHI intensifies due to episodes of high environmental pollution, it produces an increase in mortality (Arbuthnott and Hajat 2017; Chen et al. 2013; Ulpiani 2021), respiratory diseases (Gauderman et al. 2015), cardiovascular disease (Song et al. 2019) and even cancer (Pedersen et al. 2017; Zhang et al. 2022). Thus, changes in urban land cover ultimately cause microclimatic changes that —together with high rates of environmental pollution— exacerbate disease and influence the physical and mental well-being of the inhabitants of urban areas (Das and Das 2020; Shahmohamadi et al. 2011). Spain is one of the European countries showing the greatest development of artificial coverage or built surface. It is therefore wise, or urgent, to evaluate the consequences of high temperatures and pollution for adverse health effects and the quality of life of citizens. To do so, it is first necessary to identify the areas of highest building density and the high-temperature thermal spaces known as Urban HotSpot (UHS) (Amindin et al. 2021; Das and Das 2020; Sharma et al. 2021) and relate them to pollution and excess mortality risk. Recent research has concluded that exposure to heat and high concentrations of pollution is associated with adverse health effects ranging from morbidity and hospitalization to death (Heaviside et al. 2017; Shahmohamadi et al. 2011; Zhang et al. 2022). A study on the city of Birmingham reported that the areas of highest population density had higher LST and UHI values and were the areas having the highest mortality rate from disease (Tomlinson et al. 2011). A study on Paris found that high night temperatures during the summer of 2003 were associated with increased pollution and mortality (Mok et al. 2021). A broad study involving 88 large US cities concluded that the mortality rate increased by 0.5% if the previous day’s PM_10_ concentration rate had increase by around 10 µg/m^3^ (Dominici et al. 2012). A study carried out in China reported that life expectancy is approximately 5.5 years lower in the north due to a higher incidence of mortality tied to pollution (Chen et al. 2013). Finally, research involving Denmark, Austria and Italy between the years 1985 and 2005 reported increases in carcinogenic diseases associated with an increase in environmental pollution (Pedersen et al. 2017). However, these studies present some issues to review and that make our research come to provide new results that allow expanding knowledge and establishing mitigation measures in future urban developments. On the one hand, these studies use satellite images using National Aeronautics and Space Administration (NASA) Landsat 8. This unique being always orbits any point on the planet at the same time. This is a great impediment since there are numerous investigations that warn of the high spatiotemporal variability of temperatures and the phenomenon of urban heat island throughout the day. Our research has used the Sentinel 3A and 3B satellites. The first orbits during the day while the second does so at night. This allows us to analyze and study the variability of temperatures at various times of the day, obtaining more complete information. On the other hand, our research takes into account 3 variables related to urban morphology, 5 different types of environmental pollutants and 10 types of diseases. This makes it a very complete study on the relationship between high temperatures and diseases that establishes measures with respect to future urban growth that allows minimizing excess risk due to the diseases studied.

Among the different methodologies used to determine the LST, SUHI and environmental contamination, remote sensing stands out. It allows for conducting large-scale urban studies (Song et al. 2018). Thermal infrared sensors (TIRS) make it possible to carry out LST and SUHI studies, while images of environmental pollutants help determine urban pollution levels. It is crucial to analyze the relationship between urban areas, environmental pollution, LST and SUHI to understand how these variables may influence the development or rise in diseases among the population. By studying the evolution of these phenomena and their relationship with an excess risk of disease and mortality within the urban environment, researchers —and ultimately policy-makers— can promote future mitigation decisions and improve citizens’ quality of life in urban areas.

The objective of this research is to study the space–time evolution undergone by the LST, SUHI, UHS and environmental pollution in the city of Granada (southern Spain) during the year 2021, using Sentinel 3 and 5P satellite images to assess how these variables, along with the normalized difference vegetation index (NDVI), normalized difference built-up index (NDBI) and proportion of vegetation (PV), may have influenced the risk of disease (stomach, colorectal, lung, prostate and bladder cancer; dementia; cerebrovascular disease; liver disease; and suicide) among the population. To this end, the NDVI, PV, NDBI and the LST were recovered through Sentinel 3 images, and the SUHI and UHS were obtained. Next, using Sentinel 5P images, key environmental pollution data (CO, SO_2_, NO_2_, O_3_ and Aerosols) were derived. Data on excess risk of disease and mortality were obtained from the MEDEA3 mortality atlas. Finally, statistical analysis gave data correlations, and relationships between variables could be determined using the Data Panel technique. This analytical approach stands as a novel element with respect to the multiple correlation methods commonly used in research, since it allows for the incorporation of a greater number of data and variables by admitting the inclusion of the individual effects of a certain area to arrive at the global results.

The questions we intend to answer are as follows: (1) Is there a relationship between LST and SUHI, UHS, environmental contamination, NDVI, PV and NDBI? (2) Is there a relationship between these variables and the variability of excess risk due to disease and mortality? (3) Can measures be established to minimize LST and, therefore, reduce the excess risk of disease and mortality? (4) Could the results obtained prove important in future urban development?

The progress made in this research contributes to a more complete study of the relationship between LST, SUHI, UHS, environmental contamination, NDBI, NDVI, PV and risk of disease and mortality in the city of Granada. The overall aim is to envisage measures that would lessen the effects of increasing LST, SUHI and UHS and aid in decision-making by urban planners and public administrations so as to limit the health risks. Taking into account the high mortality rates and the cost that these diseases generate in the health systems of the different countries, this research should be considered as a priority starting point for new research that allows improving the quality of life of citizens. The methodology applied, entailing an open source work environment, enables one to extrapolate the results obtained to other urban areas.

## Materials and methods

### Study area

**Fig. 1:**
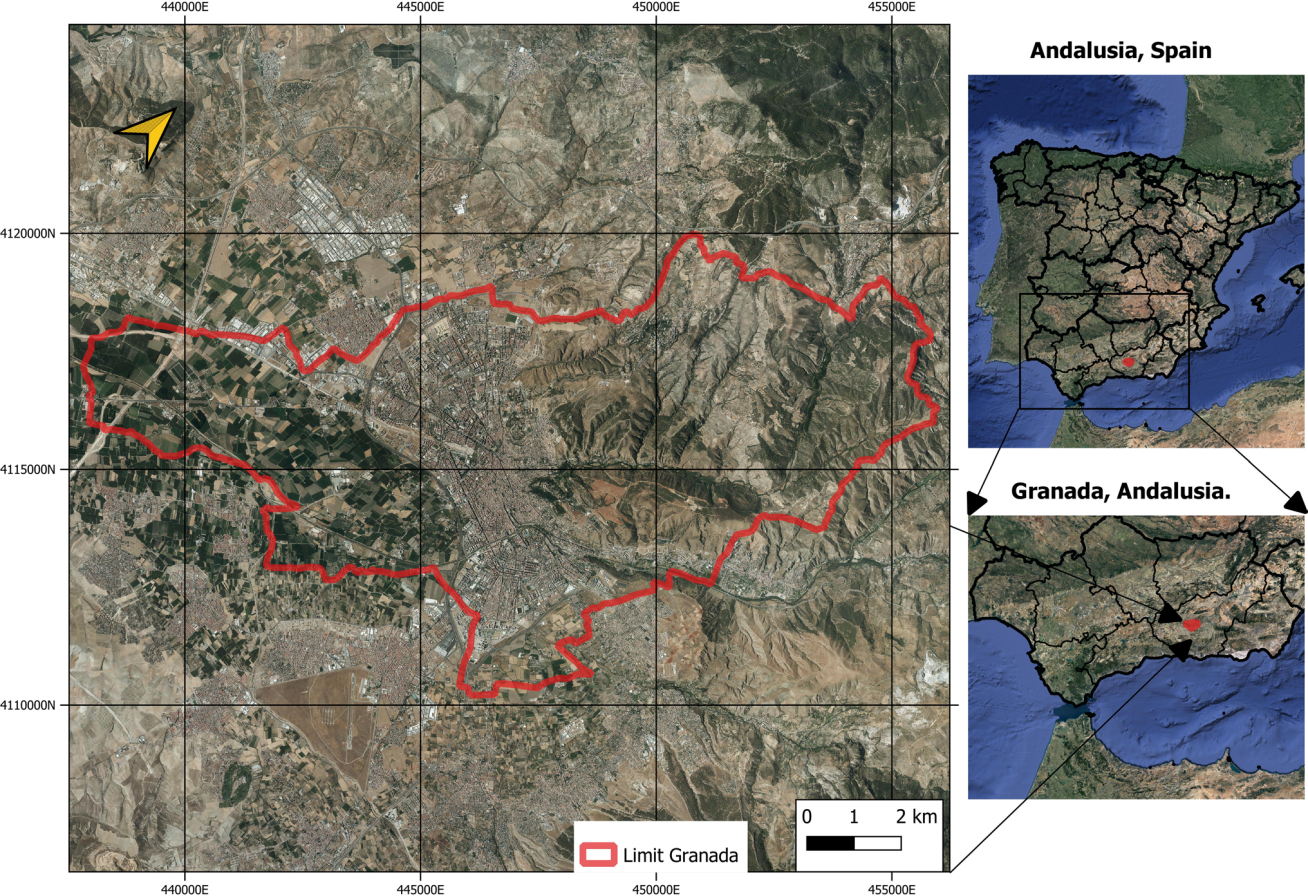
Study area: Granada, Andalusia, Spain

The area under study is the municipality of Granada, Andalusia, in southern Spain. The UTM geographic coordinates of the city are latitude 37.111741 N and longitude 03.362401 W; its altitude is 680 m above sea level (Fig. 1). It is a medium-sized city, having a population of 232,462 and an area of 880,000 km^2^. The local climate is strongly conditioned by its location at the foothills of Sierra Nevada —the second highest mountain range in Western Europe, with an average altitude of 2045 m and a maximum of 3482 m a.s.l. (Mulhacen peak)— and the proximity of the Mediterranean coast. According to the Köppen-Geiger climate classification, Granada has a transitional climate between the Mediterranean (Csa) and the cold semi-arid climate (Bsk), which implies mild, humid winters and hot, dry summers (de Castro et al. 2007). The average annual temperature fluctuates between 279.65 K in January and 298.45 K in July, with minimum temperatures in winter of 270.15 K and extreme temperatures in summer of 316.15 K. The approximate number of hours of sunshine per year is 2917 h, giving an average of 7.99 h of sunshine per day.

### Methodology

**Fig. 2:**
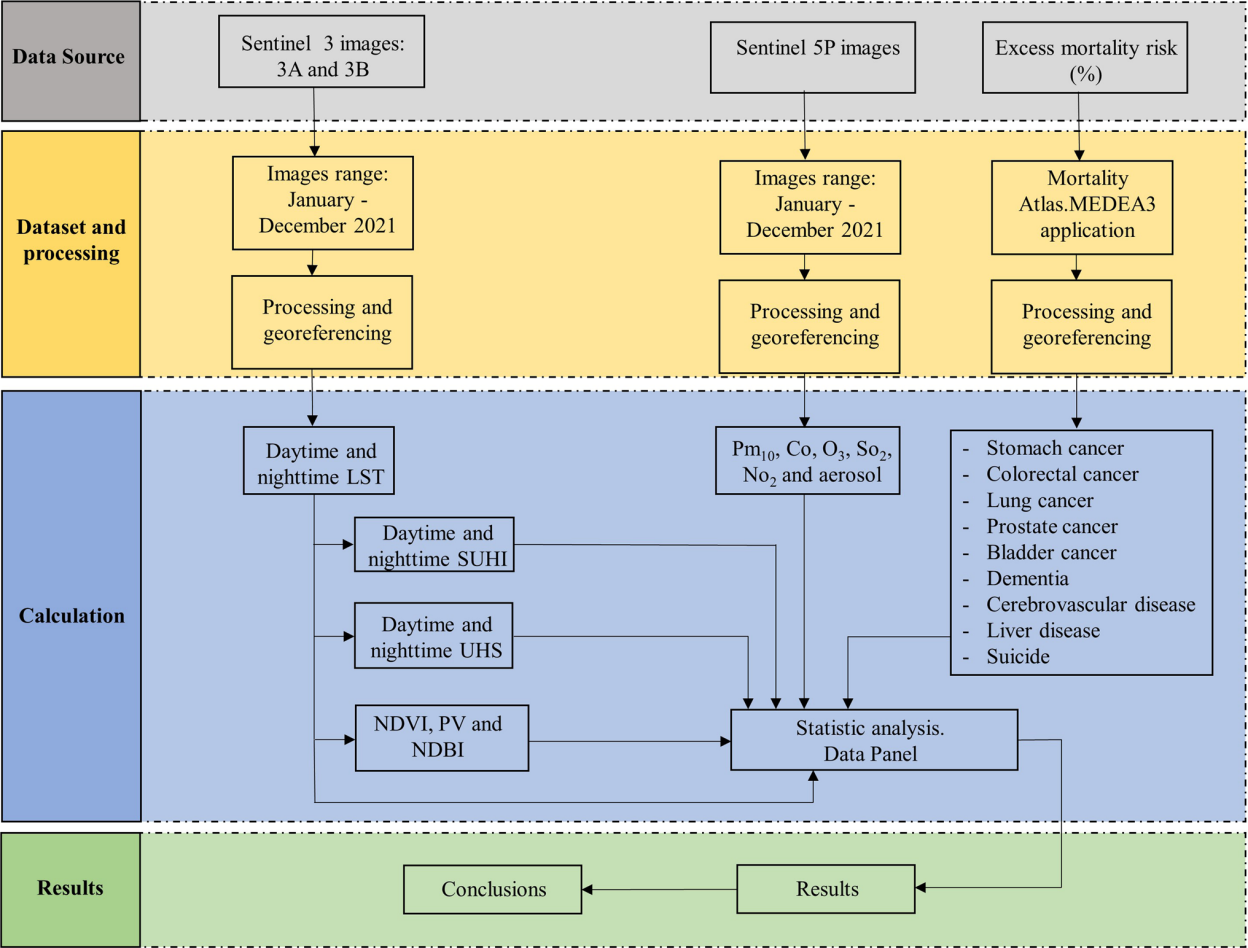
Methodology

The methodology behind the development of this research work is described in Fig. 2.

The LST, NDVI, NDBI and PV images of the area under study were acquired through the Copernicus Open Access Hub of the European Space Agency (ESA) for level 2. For the year 2021, the daytime and night LST were derived from Sentinel 3A and 3B images, while environmental pollution data came from Sentinel 5P images using the open source software QGIS, version 3.16.16 and SNAP. Data on excess risk of disease were obtained from the mortality atlas by means of the MEDEA3 application. Next, the SUHI was determined, and the diurnal and nocturnal UHS were identified using the Raster calculator tool of the indicated software. Data analysis relied on specialized software for science, STATA, version 16.

### Sentinel 3 images

To obtain the Land Surface Temperature of the Earth’s surface, Sentinel 3 features a high-resolution Land Surface Temperature Radiometer scanning instrument. The images consist of 3 thermal bands (bands S7, S8 and S9) that allow determining the LST with a resolution of 1000 m, plus six spectral bands (bands S1 to S6) with a resolution of 500 m.

Sentinel 3 tier 2 products allow direct and automatic download of the LST along with four associated parameters: normalized vegetation index (NDVI), land user/land cover (LULC), fraction vegetal (PV) and the normalized difference built-up index (NDBI).

Granada lies below the path of Sentinel 3A and 3B satellites. The usual hours of daily passage over the community are between 10:00 and 11:00 a.m. and between 9:00 and 10:00 p.m. The images chosen for the study correspond to 24 days distributed evenly between the months of January and December of the year 2021. Therefore, throughout this time interval, a total of 48 images were used, 24 corresponding to Sentinel 3A and 24 to Sentinel 3B. All have a cloudiness index of less than 15% to ensure accuracy in obtaining the LST and subsequently calculating the UHI (Table 1).

**Table 1:** Images and satellite used

Satellite/sensor	Date of acquisition (yyyymmdd)	UTC time (hh:mm)	Cloud cover (%)	Satellite/sensor	Date of acquisition (yyyymmdd)	UTC time (hh:mm)	Cloud cover (%)
3A	20,210,124	10:27	4	3A	20,210,704	22:10	2
3B	20,210,125	22:19	2	3A	20,210,704	10:53	4
3B	20,210,126	10:36	3	3A	20,210,715	22:25	7
3A	20,210,128	21:40	5	3A	20,210,716	10:42	5
3A	20,210,201	10:19	4	3A	20,210,804	22:06	5
3A	20,210,201	21:36	8	3B	20,210,804	10:13	4
3A	20,210,224	21:40	2	3B	20,210,830	21:53	0
3A	20,210,224	10:23	7	3B	20,210,830	10:36	0
3B	20,210,313	10:44	5	3B	20,210,903	21:49	8
3B	20,210,313	22:00	2	3B	20,210,903	10:32	6
3A	20,210,322	10:49	5	3B	20,210,929	22:16	2
3A	20,210,322	22:06	3	3B	20,210,929	10:59	6
3B	20,210,401	10:51	0	3A	20,211,001	22:03	5
3B	20,210,401	22:08	8	3A	20,211,002	10:19	2
3B	20,210,406	21:38	5	3A	20,211,027	22:29	1
3A	20,210,410	10:14	1	3A	20,211,028	10:46	9
3B	20,210,502	22:04	3	3B	20,211,118	11:02	0
3A	20,210,513	10:01	0	3B	20,211,120	21:27	2
3A	20,210,519	22:03	0	3B	20,211,128	10:02	4
3A	20,210,519	10:46	4	3B	20,211,130	22:08	3
3B	20,210,602	10:44	4	3B	20,211,204	22:04	2
3B	20,210,602	22:01	2	3B	20,211,204	10:47	5
3B	20,210,622	10:25	8	3A	20,211,230	21:29	3
3B	20,210,622	22:14	6	3A	20,211,230	10:12	0

Data processing consisted of reclassification at a resolution of 100 m, atmospheric correction using the Sentinel 3 Application Platform (SNAP) Toolbox and georeferencing using the ETRS89/UTM Zone 30N projection system.

### Environmental variables

The values of the environmental pollution variables O_3_, SO_2_, NO_2_, CO and aerosols on the selected dates were obtained by means of a tropospheric monitoring instrument (TROPOMI) integrated in the Sentinel-5P satellite. It was launched in 2017 and scans the Earth’s surface on a daily basis with four high-resolution spectrometers. Three cover the near-infrared ultraviolet zone with two spectral bands (270–500 nm and 675–775 nm), and one spectrometer covers the short-wave infrared. The Sentinel 5P satellite passes over the city of Granada between 11:00 a.m. and 12:00 noon. A total of 24 images were acquired through the ESA Copernicus Open Access Hub for level 2, selected for the same days as the Sentinel 3 temperature images so that the pollution and temperature values would coincide. After downloading the images, the spatial resolution of the contaminant bands was reclassified to a resolution of 100 m and georeferenced using the ETRS89/UTM Zone 30N projection system. Band reclassification was performed using the Toolbox (S3TBX) under the Sentinel Application Platform (SNAP) open source software environment, version 7.0.0.

### Land surface temperature

The Sentinel algorithms that allow for obtaining the LST are based on the concept of absorption difference (McMillin 1975), according to which it is possible to correct atmospheric effects by means of the difference between the two wavelengths of the TIRS band. Numerous studies attest to the precision and validation of these algorithms in Sentinel 3 images (Ruescas et al. 2016). The algorithm of the product SLSTR Sentinel internally includes the emissivity of the land according to the following equation (Pérez-Planells et al. 2021; Remedios and Emsley 2012):

1 $$LST={a}_{f,i,pw}+{b}_{f,i }{({T}_{11}-{T}_{12})}^{\frac{1}{\mathrm{cos}\left(\frac{\theta }{m}\right)}}+\left({b}_{f,i}+{c}_{f,i}\right) {T}_{12}$$

where LST is the temperature of the Earth’s surface in degrees K; *a*, *b* and *c* are the ground cover coefficients; and *T*_11_ and *T*_12_ are the brightness temperatures in the upper part of the atmosphere on bands 8 and 9, respectively. Subscript *f* corresponds to the vegetation fraction; *i* denotes vegetation type; and *pw* is the atmospheric water column vapour content. In turn, *θ* is the zenith angle of view of the satellite located in the metadata file, and *m* is a dependent variable of θ (Remedios and Emsley 2012; Yang et al. 2020b).

Sentinel 3 products with processing level 2 allow direct determination of the LST, as the application of the algorithm (1) is performed internally by the open-source Sentinel Application Platform (SNAP) software. After determining the LST of the city, the data was exported in raster images using the software QGIS.

### Surface urban heat island

According to the literature, the SUHI is obtained by means of the temperature difference detected between measurements made simultaneously in the urban area and the rural area (Oke 1987). Therefore, the SUHI can be determined according to Eq. 2:

2 $$SUHI ={LST}_{urban}- {LST}_{rural}$$

The urban LST values correspond to the average values of the pixels located within the urban area. The rural area chosen to derive the SUHI through the temperature differences from the urban area corresponds to where the Spanish State Meteorological Agency (AEMET) has a rural weather station, 15–16 km outside the city, and there are no paved areas within a radius of 1000 m. There are numerous SUHI studies using the so-called local climate zones (LCZ) (Anjos et al. 2020; Das and Das 2020; Wang and Ouyang 2017) as typologies of landscapes at a local scale located within and around the cities. In this research, this typology has not been chosen since, in order to minimize the impact of the mismatch of the definitive resolution of the LCZs, they must have a size of at least 1 km in diameter (Bechtel et al. 2019). The city of Granada, being a small city, would have few LCZs with that minimum size. Using the raster calculator command of QGIS software and the exported Landsat images, the SUHI of Granada was determined by Eq. 2

### Urban hotspots

Hot spots are identified based on the LST within the study area. They are zones of variable size found within places giving the highest temperatures, and they are usually considered as uncomfortable for human activities. They can be determined using the following formula (Guha 2017; Sharma et al. 2021):

3 $$LST> \mu +2* \sigma$$

where *µ* and $\sigma$ are respectively the mean value and the standard deviation of the LST of the zone in ºC. Areas that present urban zones with LST values above the mean and, with a confidence interval greater than 95%, can thus be determined.

### Mortality Atlas: MEDEA3

The data on deaths from disease and the excess risk of disease and mortality for the city of Granada were obtained from the mortality atlas of the MEDEA3 research group of the University of Valencia (Spain). It shows the main results of the research project “Socioeconomic and environmental inequalities in the geographical distribution of mortality in large cities of Spain (1996–2015): MEDEA3” financed by the Carlos III Health Institute and co-financed by the European Development Fund Regional (ERDF) of the European Union (MEDEA3 2022). The diseases selected for this research have been chosen based on two variables of great importance and social repercussion: (1) They are those that have produced the greatest number of deaths in the city investigated in the year 2021. (2) They are those that present a greater cost of treatment in public hospitals financed by the taxes of all citizens. Therefore, these are the ones with the greatest social, family and economic repercussion.

### Strategy of analysis

Panel data refers to a statistical analysis that combines a temporal dimension (time) with a cross-sectional dimension (data or values). This method is often cited in the literature and involves the use of multiple regression models (Alcock et al. 2015; Chen et al. 2011; Fang and Tian 2020), which allows for a larger amount of data to be included than traditional methods. The method is compatible with three calculation options: ordinary squares method (OSM), generalized least squares (GLS) and intragroup estimation method (IEM) (Labra 2014). To know which of the three is best to use, the following steps must be carried out (Chen et al. 2011): (1) Using the Hausman test, determine if the effects of the analysis are fixed or random. This allows the method to determine different hypotheses about the behaviour of the residuals of statistical analysis. (2) Evaluation of the model using the Wooldridge and Wald tests. Both phases will indicate the most appropriate method to use (Seto and Kaufmann 2003). Statistical analysis was performed with STATA software, version 16. For our research, after carrying out the tests, the IEM method with random effects was used according to Eq. 4:

4 $${Y}_{it}= \beta {X}_{it}+\left({\alpha }_{i}+{\mu }_{it}\right)$$

where ${\mu }_{it}$ is the error of the model, ${\alpha }_{i}$ represents the individual effects, *X*_*it*_ are explanatory variables, *β* is an independent variable, *t* = time and *i* = individual.

This method allows for a greater amount of data in the analysis, thereby increasing the degree of freedom, while reducing inconvenient collinearity between the variables. By taking into account individual effects, the final function obtained for the set of individuals is totally different from the one that would have been obtained using other statistical techniques. It is assumed that individual effects are not reflected in the explanatory variables of the model; instead, they contribute to the error term. Sampling for statistical analysis was performed through graphic documentation that includes the results obtained. To do this, a mesh with 400 reference points was drawn on the area under study. Next, the values and indices, LST, SUHI, contamination and excess risk due to disease for each point were obtained. These values were entered into the data analysis software used to obtain the results using the IEM Eq. 4 method. On the other hand, and as the excess risk variable due to disease is determined by each urban area of the city and in order to be able to analyze the data statistically, the mean value of the rest of the indices and variables analysed (LST, SUHI, UHS, NDVI, NDBI and PV) for each urban area.

## Results

### Space–time evaluation of spectral indices (NDVI, NDBI and PV)

**Fig. 3:**
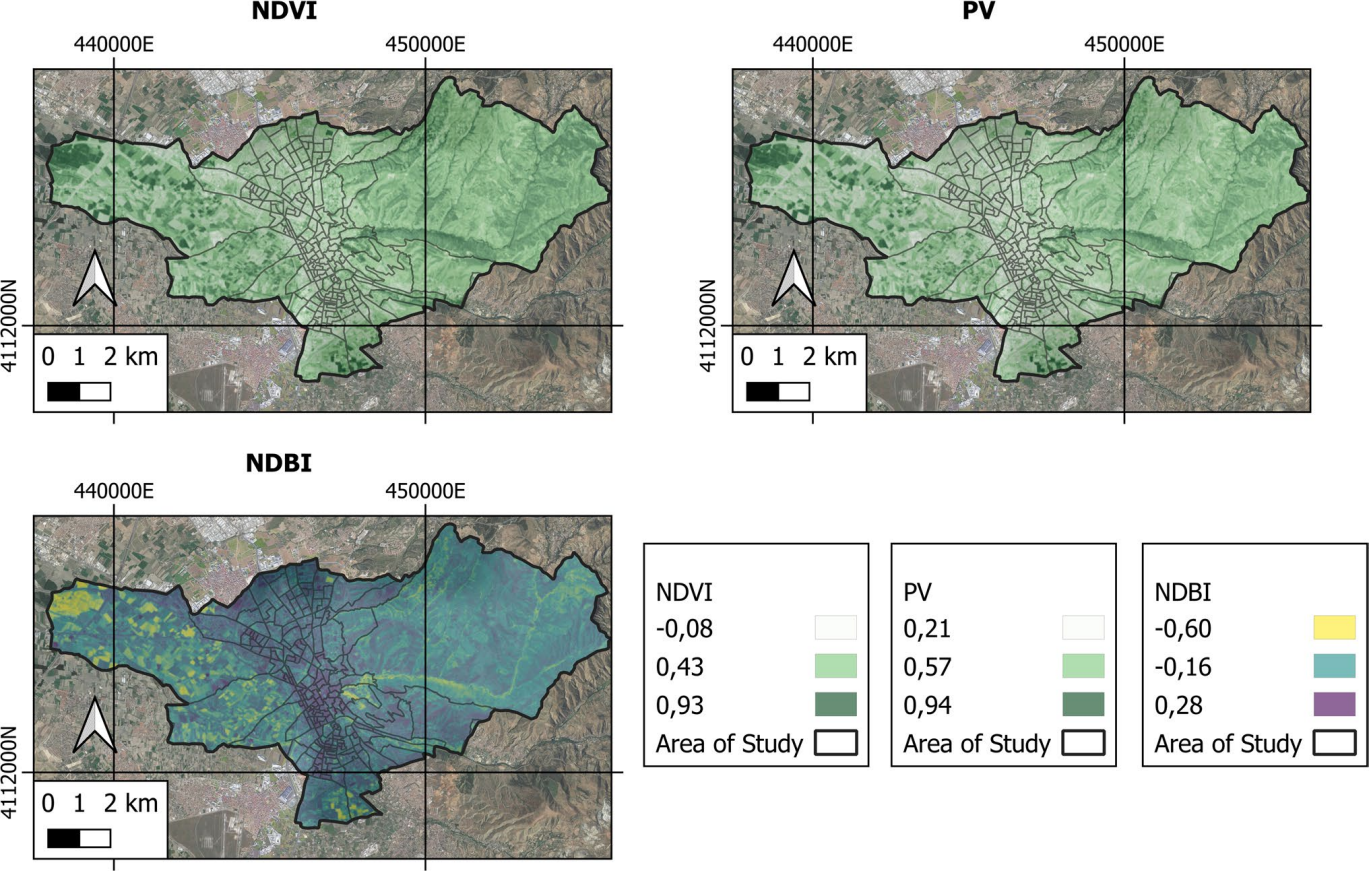
NDVI, PV and NDBI indices for the area under study

The space–time analysis of the NDVI, NDBI and PV spectral indices for the year 2021 are represented in Fig. 3.

The NDVI can be interpreted as a measure of the amount and state of vegetation on the surface. The PV index establishes the proportion that exists in a given area between the areas with vegetation and the built-up areas. Finally, the NDBI allows carrying out the determination of built-up or developing areas. In the city of Granada, the NDVI index oscillates between the maximum value of 0.934 and the minimum value of − 0.077, the mean being 0.411. The NDBI index ranges between the maximum value of 0.285 and the minimum of − 0.602, with a mean value of − 0.082. Lastly, the PV index oscillates between the maximum of 0.935 and the minimum of 0.212, with a mean value of 0.506. The highest values for the NDVI and PV indices are found in rural areas, while the lowest ones pertain to urban areas. Contrariwise, the highest values for the NDBI index are found in urban areas, as opposed to the lowest values, found in rural areas. This is due to the fact that rural areas have high percentages of green areas in good condition compared to urban areas where built-up or developing areas are higher than small green areas. This circumstance is corroborated by the values obtained for the NDBI index. In this way, it presents the highest values in urban areas as opposed to the lowest values found in rural areas. Therefore, the greater the urban development of an area, the higher the NDBI index values and the lower the NDVI and PV index values, and vice versa.

### TST validation and contamination

The LST and the environmental contamination obtained through satellite images require a verification process that allows the results obtained to be validated. In the last decades, the comparison method of the LST and the contamination obtained by means of satellite images with the environmental temperatures and the contamination of the meteorological stations is gaining importance as a data validation system (Avdan and Jovanovska 2016; Gallo et al. 2011; Liu and Zhang 2011; Mukherjee and Singh 2020; Rongali et al. 2018). This consists of comparing the LST and contamination recovered with the temperature and environmental contamination obtained from meteorological stations or temperature probes located near the ground (1–2 m). Through this system, the statistical variables take on importance: *R*^2^ regression coefficient, root mean square error (RMSE) and the mean error bias (MBE). In our research, and in order to validate the TST and contamination obtained by means of Sentinel 3 and 5P satellite images, the values of environmental temperature and contamination of the stations of the State Meteorological Agency (AEMET) have been acquired during the hours of passage of the satellites located in the city of Granada: Bus station and Congress Palace.

In general, the LST and environmental contamination values of Sentinel 3 and 5 are higher than those obtained by the weather station. The validation results obtained are the following: LST: *R*^2^ = 0.931; RMSE = 2.341 ºC and MBE =  − 0.128 ºC; O3: *R*^2^ = 0.924; RMSE = 0.023 µm/m^3^ and MBE = 0.014 µm/m^3^; CO: *R*^2^ = 0.912; RMSE = 0.012 µm/m^3^ and MBE = 0.024 µm/m^3^; SO_2_: *R*^2^ = 0.950; RMSE = 0.00011 µm/m^3^ and MBE = 0.00032 µm/m^3^; NO_2_: *R*^2^ = 0.913; RMSE = 1 × 10^−6^ µm/m^3^ and MBE = 2 × 10^−7^ µm/m3; Aerosol: *R*^2^ = 0.951; RMSE = 0.012 µm/m^3^ and MBE =  − 0.022 µm/m^3^.

### Spatiotemporal evaluation of the LST and SUHI

**Fig. 4:**
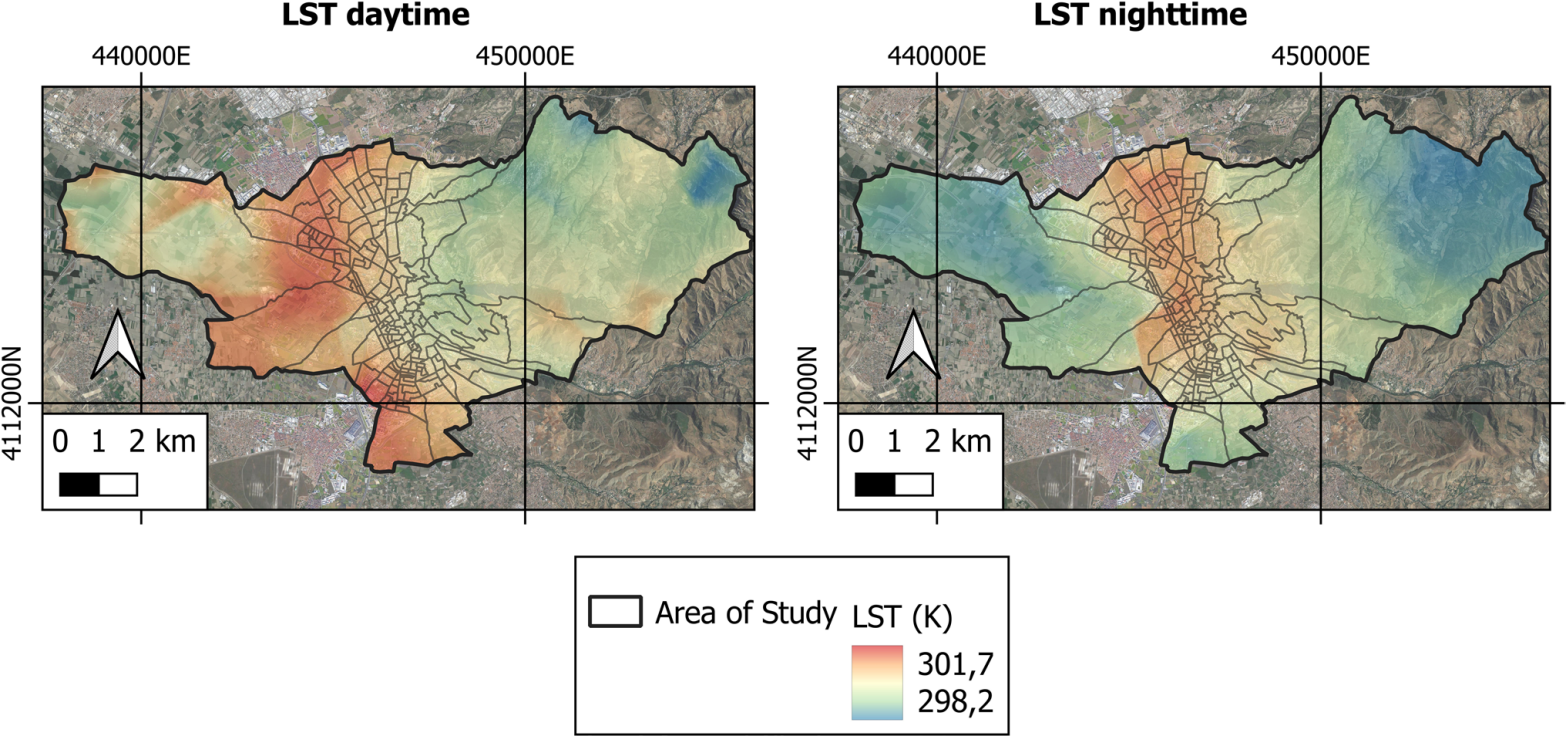
Variability of the diurnal and nocturnal LST of the area under study

**Fig. 5:**
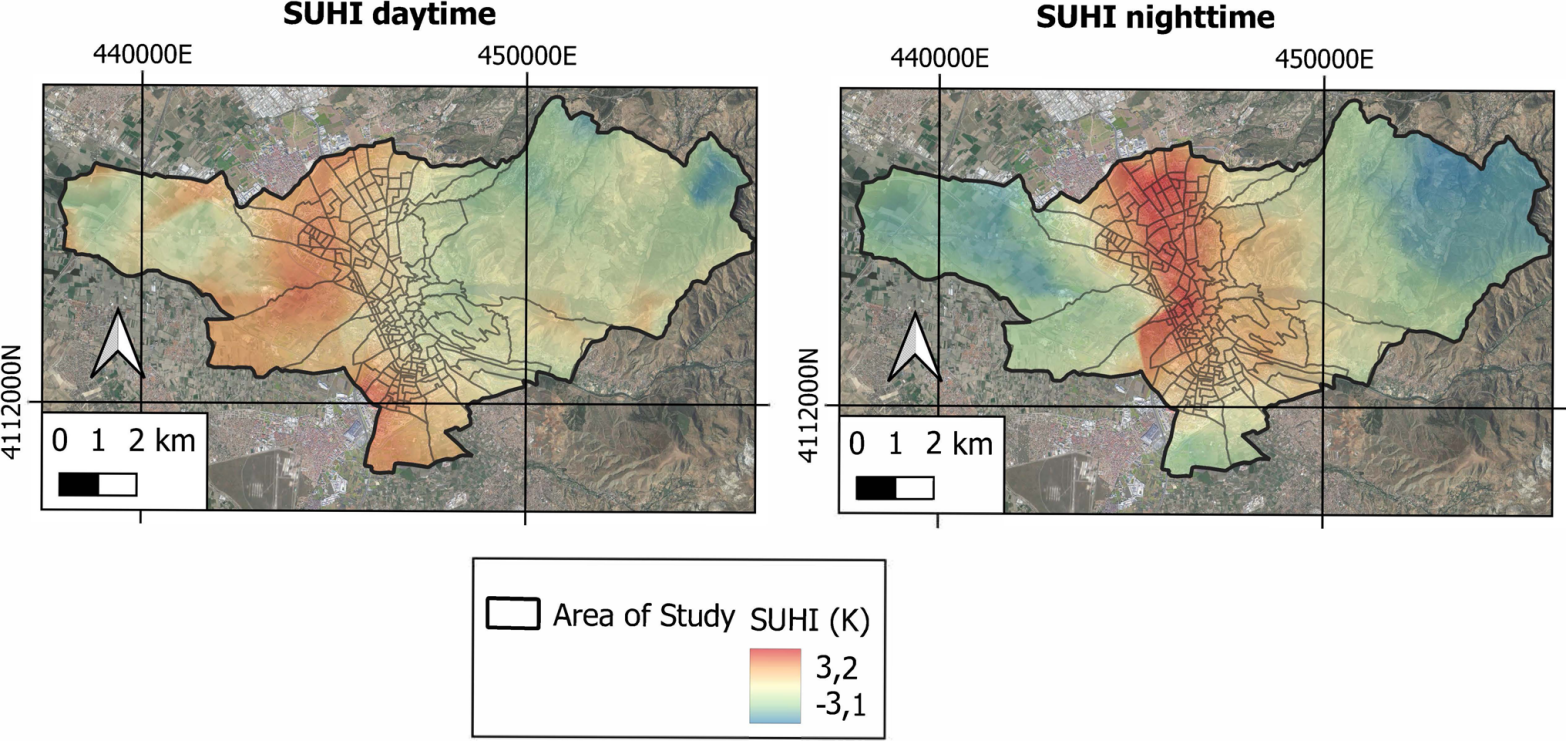
Variability of day and night SUHI in the study area

Figures 4 and 5 show the space–time analysis of the LST and the SUHI of the metropolitan area under study.

In the city of Granada, the daytime LST oscillated between a maximum value of 301.61 K and a minimum value of 298.23 K, giving an average value of 300.12 K. In contrast, the night-time LST ranged from a maximum value of 288.22 K to a minimum of 284.91 K, the average value being 286.34 K. During the mornings the highest values of LST correspond to rural areas, whereas the lowest LST values occur in urban areas. Yet the highest values of nocturnal LST are reported in the city. The daytime SUHI was found to range between a maximum value of 0.71 K and a minimum of − 2.82 K, with a mean value of − 0.33 K. The nocturnal SUHI, in turn, oscillated between 3.22 K (maximum) and − 0.12 K (minimum), the mean value being 1.33 K. Hence, the highest values of daytime SUHI pertain to rural areas, the lowest ones occurring in urban areas. To the contrary, the highest values of nocturnal SUHI are reported in urban areas, and the lowest values in rural areas.

This is due to the fact that during the mornings, rural areas without vegetation receive high doses of solar radiation, as opposed to urban areas that receive less radiation due to the shadows generated by buildings and vegetation in green areas. Therefore, the former heat up faster than the latter. On the contrary, and once the sun goes down, rural areas cool quickly due to the low thermal inertia of the ground, while urban areas built with materials with high thermal absorption (bricks, asphalt, concrete) gradually release heat into the atmosphere slowly. Secondly, the high density of buildings and population in Granada, especially in the downtown areas, means greater energy consumption. It is important to note that the energy consumed by air conditioning systems is released into the environment, further raising the urban temperature in those areas.

### UHS identification

**Fig. 6:**
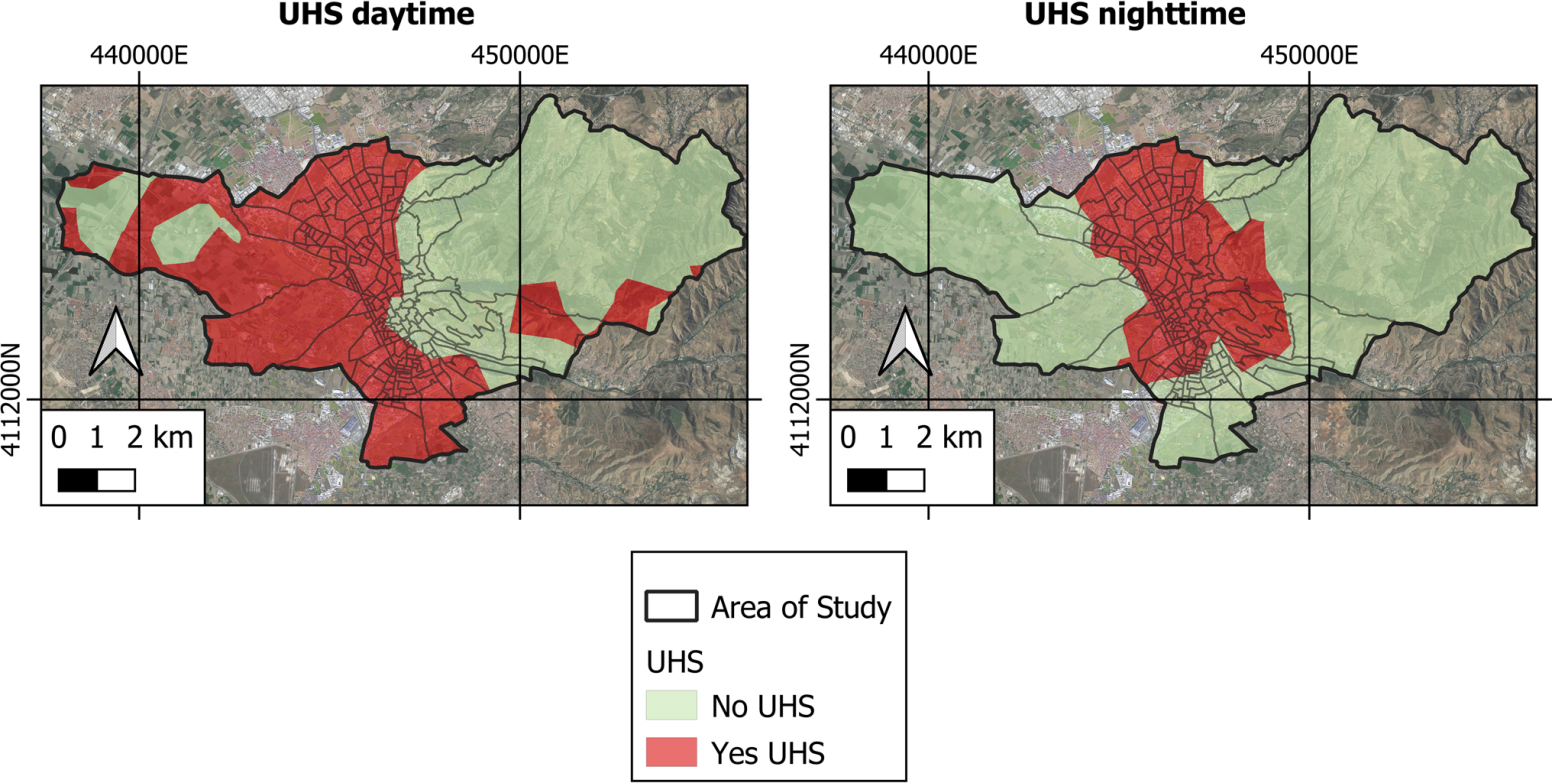
Variability of day and night UHS in the study area

Figure 6 shows the space–time analysis of the UHS of Granada for the year 2021. Table 2 offers the critical values for determining UHS as well as their extension, and the percentage of occupation with respect to the total area studied.

**Table 2:** Average and critical LST for the determination of day and night UHS, along with the occupation of these zones

	Mean LST (K)	SD (K)	Non UHS (K)	UHS (K)	UHS (ha)	UHS (%)
Daytime	300.12	0.74	< 301.60	≥ 301.60	4166	47.32
Night-time	286.34	0.81	< 287.96	≥ 287.96	2150	24.43

*SD* standard deviation, *UHS* urban hot spot

The area of daytime UHS is 4166 ha, which represents 47.32% of the total area of the municipality of Granada. The nocturnal UHS is 2150 ha, representing 24.43% of the total area. The daytime UHSs are found in the peripheral urban areas and in rural areas, where the highest daytime LST values are also found. In contrast, overnight UHSs are concentrated in urban areas as opposed to rural areas. This is due to the fact that at night, the highest LST values are concentrated in urban areas.

### Environmental pollution

**Fig. 7:**
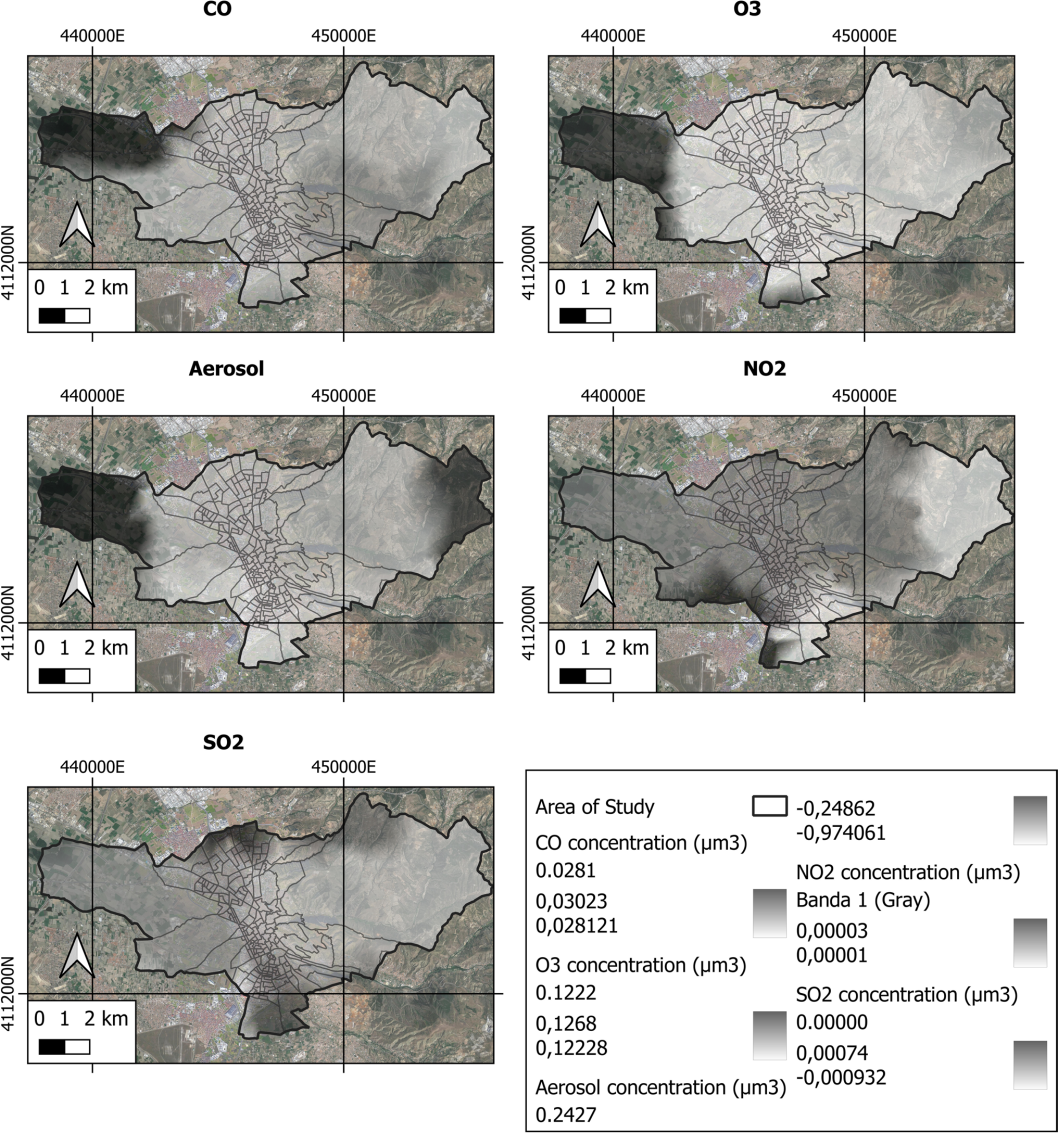
Variability of environmental pollutants in the area under study

Figure 7 shows the space–time analysis of environmental pollutants (O_3_, CO, SO_2_, NO_2_ and aerosols) in Granada during the year 2021 as obtained by Sentinel 5P. Table 3 presents the measures of central tendency and dispersion of each variable studied.

**Table 3:** Dispersion measures of excess risk due to diseases

	Max (%-µm/m^3^)	Min (%-µm/m^3^)	Mean (%-µm/m^3^)	SD (%-µm/m^3^)
Stomach cancer	73.41	− 29.41	3.67	14.71
Colorectal cancer	81.48	− 32.14	4.57	17.05
Lung cancer	95.35	− 297	4.58	16.86
Prostate cancer	46.72	− 26.68	3.88	11.58
Bladder cancer	96.96	− 31.92	4.53	18.43
Dementia	49.61	− 19.43	3.92	10.78
Cerebrovascular disease	67.08	− 36.40	4.05	14.71
Liver disease	201.31	− 52.02	5.98	33.39
Suicide	92.87	− 156.14	2.94	20.91
O_3_	0.1268	0.1222	0.1230	0.0013
CO	0.0302	0.0281	0.0287	0.0005
SO_2_	0.00074	0.00000	0.00034	0.00030
NO_2_	$3\times {10}^{-5}$	$1\times {10}^{-5}$	$1.8\times {10}^{-5}$	$4.5\times {10}^{-6}$
aerosols	0.2427	0.9727	0.7312	0.2181

Results in % for disease excess and in µm/m^3^ for concentrations

*SD* standard deviation

Figure 7 indicates that the highest concentrations of pollutants SO_2_ and NO_2_ occur in urban areas as opposed to the lowest concentrations, which occur in rural areas. In contrast, the highest concentrations of pollutants O_3_, CO and aerosols occur in rural areas; the lowest concentrations are seemed in urban areas. In recent decades, air pollution has become a very serious problem for the city of Granada. In fact, the city is currently rated as the 3rd most polluted city in Spain, behind Madrid and Barcelona, which have many more inhabitants. According to world standards, the city’s air quality is poor, posing a serious environmental and health problem for the population. It is known that 60 to 70% of total emissions are caused by industry and urban transport. Urban development and a poor public transport system have led to a growing number of vehicles circulating in the city. The problem is aggravated by topographical factors (Sierra Nevada located to the east) and climatological factors (high solar radiation and frequent thermal inversion phenomenon) that favour the photochemical transformation of organic compounds, producing smog and tropospheric ozone.

### Disease

**Fig. 8:**
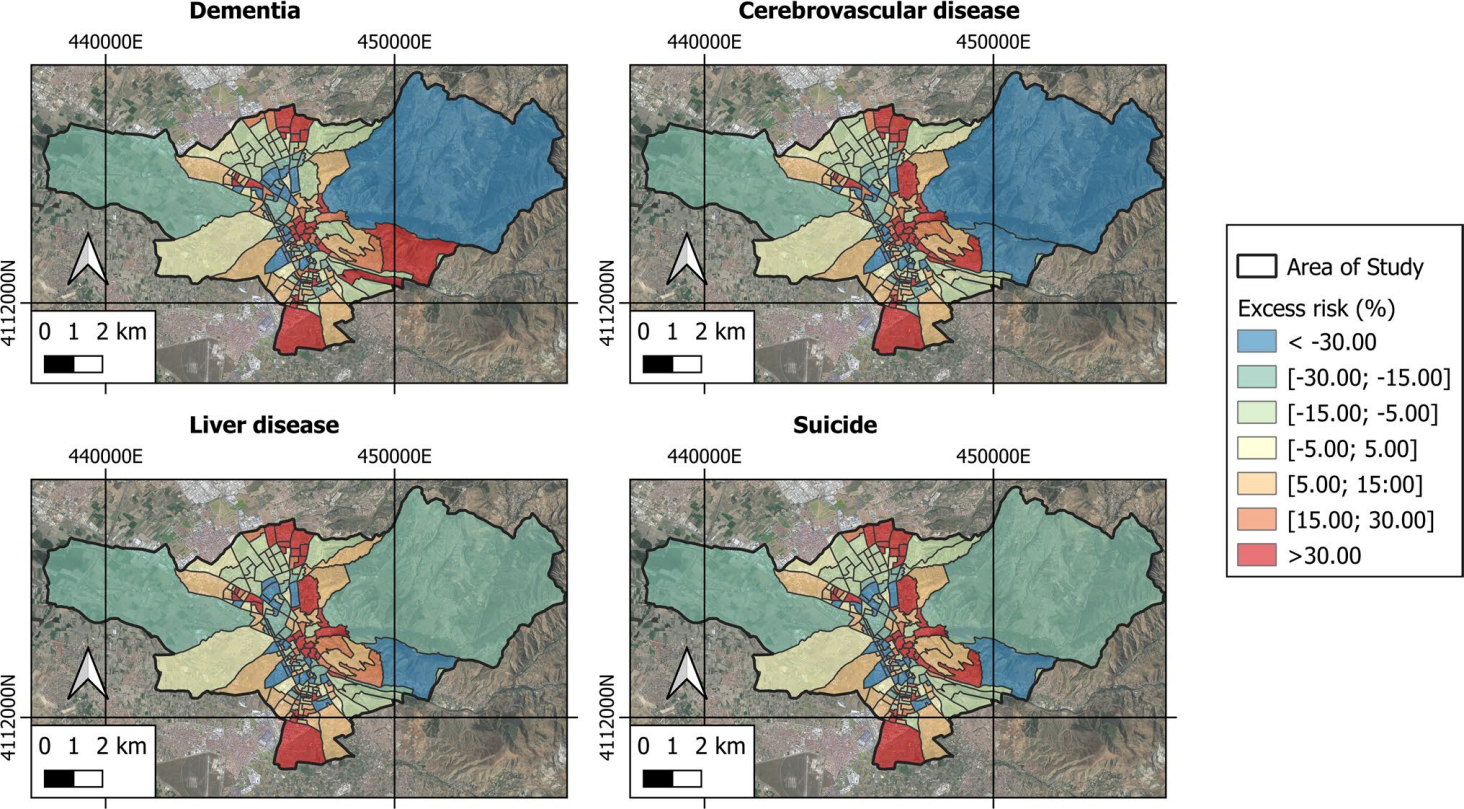
Excess risk of different types in the area under study

**Fig. 9:**
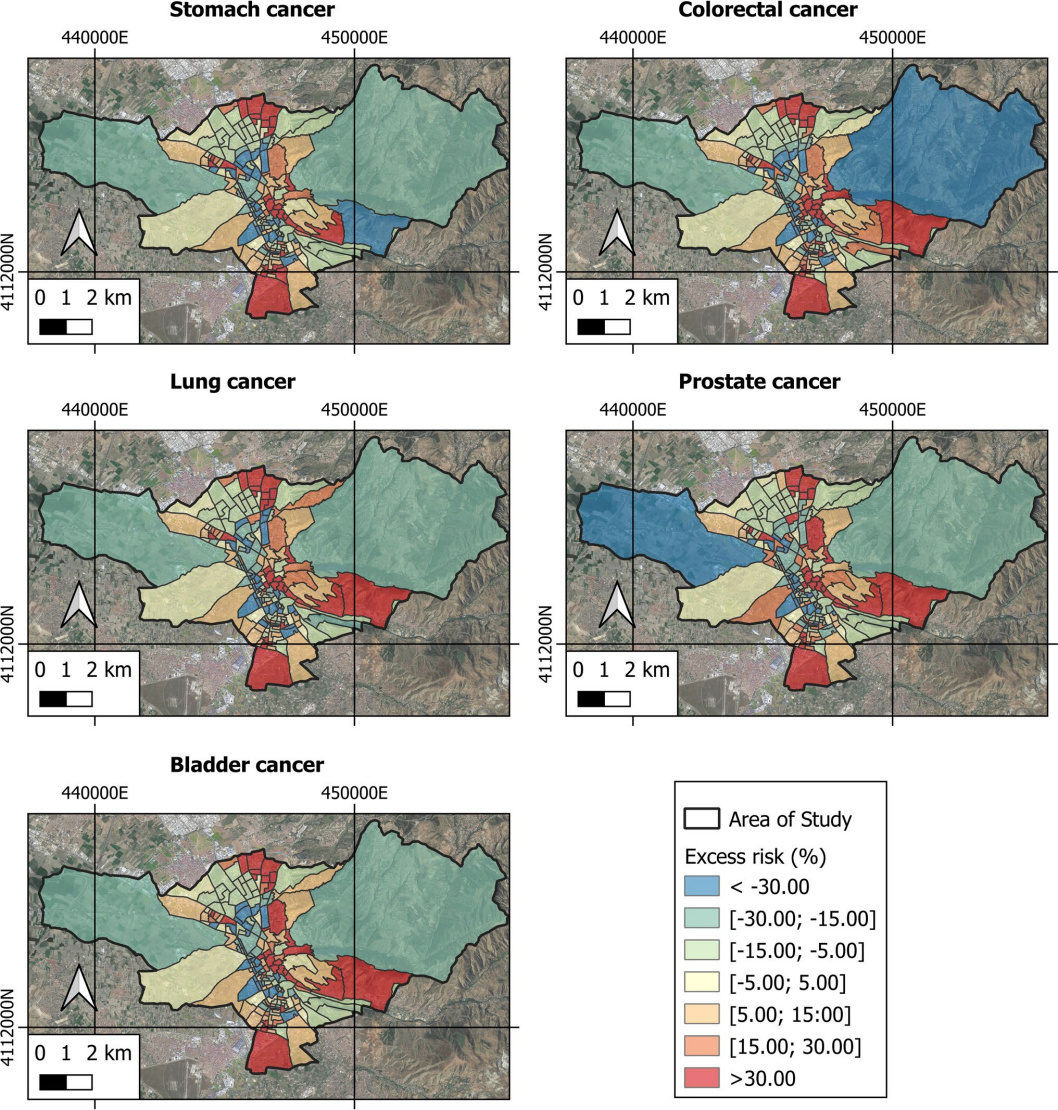
Excess risk of the different types of cancer in the area under study

Figures 8 and 9 show the spatiotemporal analysis of the excess risk of the diseases and types of cancer investigated, obtained from the MEDEA3 mortality atlas. Table 3 presents the measures of central tendency and dispersion of each variable studied. The highest risk excesses of contracting a disease are represented with red shades while the lowest risk excesses are represented with blue and green shades.

Figures 8 and 9 show how the excess risk of the diseases and types of cancer studied have higher values in urban areas as opposed to rural areas, where the excess risk takes on negative values.

### Statistical analysis

#### Relationship between LST and SUHI, UHS, NDVI, NDBI, PV and environmental contamination

Our statistical analysis used the Data Panel method to determine the relationships between the LST and the SUHI, UHS, NDVI, NDBI, PV and environmental contamination of the study area; two analyses were carried out, one with the daytime values and the other with the night-time values for LST, SUHI and UHS. First, the Pearson correlation coefficient was determined, and then the Data Panel was developed, applying the generalized least squares (GLS) method through Eq. 4. The results are given below (Table 4).

**Table 4:** Statistical analysis results LST, SUHI, UHS, NDVI, NDBI, PV and contamination

	Daytime				Night-time			
	Pearson coefficient	*P* value	*β*	Sd	Pearson coefficient	*P* value	*β*	Sd
SUHI	0.8991	0.000***	0.03654	0.00005	0.8432	0.000***	0.07089	0.00024
UHS	0.7385	0.000***	0.00039	0.00004	0.7292	0.000***	0.00084	0.00032
NDVI	− 0.2784	0.317	0.21657	0.33221	− 0.4114	0.073	1.11263	0.65423
PV	− 0.2804	0.063	− 0.5712	0.4890	− 0.4185	0.029*	− 1.82302	0.96303
NDBI	0.1045	0.000***	− 0.1613	0.0472	0.3948	0.013*	0.91538	0.09307
O_3_	0.1322	0.016*	18.970	7.9994	− 0.3631	0.000***	− 59.3608	14.4453
SO_2_	0.4970	0.000***	27.328	4.8970	− 0.1150	0.241	7.72550	8.8431
NO_2_	0.2013	0.003**	2142.01	709.580	0.5252	0.019*	8350.01	1281.35
CO	− 0.1591	0.000***	− 29.140	18.108	− 0.4564	0.005**	− 25.5132	32.700
Aerosol	− 0.0717	0.049*	− 0.0615	0.0308	− 0.0446	0.423	0.11527	0.05573
*R*^2^		0.372				0.994		
*F*		14.112				48,482		
Prob > chi^2^		0.000				0.000		

*β* coefficient, *Sd* standard deviation, *R*^2^ linear regression coefficient, *F F* statistic

Robust standard errors: *p < 0.05; **p < 0.01; ***p < 0.001

The basic unit of analysis in this section is the diurnal and nocturnal LST variable. The first presents strong positive correlations with diurnal SUHI (0.8991), diurnal UHS (0.7385), SO_2_ (0.4970) and NO_2_ (0.2013) and an inverse relationship with the NDVI (− 0.2784) and PV (− 0.2804) indices. The nocturnal LST variable presents strong positive correlations with nocturnal SUHI (0.8432), nocturnal UHS (0.7292), NDBI (0.3948) and NO_2_ (0.5252), as well as an inverse relationship with NDVI (− 0.4114), PV (− 0.4185) and pollutants O_3_ (− 0.3631) and CO (− 0.4564).

Analysis using the Data Panel technique reports a statistically significant and positive relationship above 99% between the diurnal LST, diurnal SUHI, diurnal UHS, NDBI and SO_2_ variables, whereas the relationship is negative with the CO variable. The relationship becomes positive and 99% with the variable NO_2_, positive and 95% with the O_3_ variable and negative with the aerosol’s variable. The values of *R*^2^, *F* statistic and Prob > chi^2^ show good concordance between the dependent variable and the independent ones used, with an adjustment level greater than 99% significance, since Prob > chi^2^ = 0.000.

With respect to the nocturnal LST variable, a statistically significant and positive relationship above 99% appears with the nocturnal SUHI and the nocturnal UHS; it is negative with the O_3_ variable. The relationship becomes negative and 99% with the CO variable, 95% and positive with the NDBI and NO_2_ variables and negative with the PV variable. The values of *R*^2^, *F* statistic and Prob > chi^2^ obtained show good agreement between the dependent variable and the independent ones used, the adjustment level being greater than 99% significance as Prob > chi^2^ = 0.000.

#### Relationship between the diseases studied and the rest of the variables

The results of our analysis of excess risk of disease and the rest of the variables analysed are shown in Tables 5 and 6.

**Table 5:** Statistical analysis daytime LST, SUHI, and UHS, plus NDVI, NDBI, PV and contamination results

	Stomach cancer	Colorectal cancer	Lung cancer	Prostate cancer	Bladder cancer	Dementia	Cerebrovascular disease	Liver disease	Suicide
LST daytime	(0.1341) 0.026*	(0.1165) 0.028*				(0.1294) 0.045*	(0.0562) 0.049*		(0.1181) 0.039*
SUHI daytime	(0.1384) 0.001**	(0.1194) 0.002**	(0.0862) 0.028*	(0.0545) 0.002**	(0.1150) 0.009**	(0.1320) 0.003**	(0.0593) 0.004**	(0.1123) 0.019**	(0.1222) 0.005**
UHS daytime	(0.1465) 0.003**	(0.0913) 0.003**	(0.0697) 0.034*	(0.2473) 0.000***	(0.0620) 0.018*	(0.0734) 0.006**	(0.0169) 0.001**	(0.0482) 0.037*	(0.0716) 0.010**
NDVI			(− 0.1054) 0.046*					(-0.1170) 0.036*	
PV									
NDBI	(0.0450) 0.034*	(0.0392) 0.012*	(0.0062) 0.013**	(0.0294) 0.006**	(0.0083) 0.024*	(0.0697) 0.013*	(0.0770) 0.002**	(0.0216) 0.033*	(0.0223) 0.013**
O_3_			(0.0605) 0.031*					(0.0523) 0.031*	
SO_2_	(0.350) 0.000***	(0.2732) 0.001**	(0.3533) 0.000***	(0.1603) 0.013*	(0.3544) 0.000***	(0.2651) 0.002**	(0.1973) 0.004**	(0.3874) 0.000***	(0.3352) 0.000***
NO_2_	(0.1212) 0.036*	(0.1345) 0.037*	(0.2291) 0.001**	(0.1362) 0.034*	(0.1732) 0.010*		(0.1992) 0.016*	(0.2077) 0.002**	(0.1712) 0.014**
CO									
Aerosol			(0.1460) 0.034*					(0.1281) 0.037*	
*R*^2^	0.351	0.283	0.402	0.283	0.291	0.270	0.302	0.414	0.371
*F*	5.34	4.44	7.50	6.50	5.59	3.69	4.34	6.98	5.55
Prob > chi^2^	0.0000	0.0000	0.0000	0.0000	0.0000	0.0002	0.0000	0.000	0.0000

*R*^2^ Linear regression coefficient, *F F* statistic

Robust standard errors: *p < 0.05; **p < 0.01; ***p < 0.001

**Table 6:** Statistical analysis night-time LST, night-time SUHI, night-time UHS, NDVI, NDBI, PV and morbidity results

	Stomach cancer	Colorectal cancer	Lung cancer	Prostate cancer	Bladder cancer	Dementia	Cerebrovascular disease	Liver disease	Suicide
LST night-time									
SUHI night-time									
UHS night-time									
NDVI									
PV									
NDBI	(0.0451) 0.034*	(0.0394) 0.012*	(0.0063) 0.013**	(0.0291) 0.006**	(0.0081) 0.024*	(0.0691) 0.013*	(0.0770) 0.002**	(0.0213) 0.033*	(0.0225) 0.013**
O_3_			(0.0605) 0.031*					(0.0523) 0.031*	
SO_2_	(0.3503) 0.000***	(0.2730) 0.001**	(0.3532) 0.000***	(0.1600) 0.013*	(0.3545) 0.000***	(0.2652) 0.002**	(0.1971) 0.004**	(0.3872) 0.000***	(0.3352) 0.000***
NO_2_	(0.1215) 0.036*	(0.1342) 0.037*	(0.2294) 0.001**	(0.1363) 0.034*	(0.1733) 0.010*		(0.1992) 0.016*	(0.2071) 0.002**	(0.1712) 0.014**
CO									
Aerosol			(0.1462) 0.034*					(0.1280) 0.037*	
*R*^2^	0.312	0.271	0.375	0.203	0.332	0.264	0.262	0.380	0.341
*F*	4.41	3.70	5.95	2.51	5.01	3.47	3.45	6.12	5.01
Prob > chi^2^	0.0000	0.0002	0.0000	0.0073	0.0000	0.0004	0.0004	0.0000	0.0000

*R*^2^ Linear regression coefficient, *F F* statistic

Robust standard errors: *p < 0.05; **p < 0.01; ***p < 0.001

The basic unit of analysis in this section is the excess risk of the different diseases investigated. Excessive risk due to disease presents strong positive correlations with the SO_2_ and NO_2_ variables and intermediate correlations with the SUHI, UHS and NDBI. The Data Panel results report a statistically significant and positive relationship above 99% between SO_2_ and excess risk of all the selected diseases. This relationship becomes positive at 99% with the daytime SUHI and daytime UHS variables; positive and 95–99% with the NDBI variable and positive and 95% with the diurnal LST variable and NO_2_. Lung cancer and liver disease are seen to present a statistically significant and positive relationship of 95% with the variables aerosols and O_3_. The values of *R*^2^, *F* statistic and Prob > chi^2^ obtained indicate a good concordance between the dependent variable and the independent ones used, with an adjustment level greater than 99% significance since Prob > chi^2^ = 0.000.

The excess risk of disease presents strong positive correlations with SO_2_ and NO_2_ variables and intermediate correlation with the NDBI. The results of statistical analysis using the Data Panel technique signal a statistically significant and positive relationship above 99% between SO_2_ and the excess risk of all the diseases. It becomes 95–99% with the NDBI variable. Lung cancer and liver disease present a statistically significant and positive relationship of 95% with the aerosol and O_3_ variables. This circumstance makes sense, given that the NDBI, NDVI and PV variables did not present variations between day and night readings, while the pollution values only have day readings. No statistically significant relationships were seen for the LST, SUHI and UHS variables. The values of *R*^2^, *F* statistic and Prob > chi^2^ obtained show good concordance between the dependent variable and the independent ones used, the adjustment level being greater than 99% significance as Prob > chi^2^ = 0.000.

#### Statistical analysis between environmental variables and excess diseases risk

The results of our analysis of environmental variables and excess disease risk are shown in Table 7.

**Table 7:** Statistical analysis night-time LST, night-time SUHI, night-time UHS, NDVI, NDBI, PV and morbidity results

	Stomach cancer	Colorectal cancer	Lung cancer	Prostate cancer	Bladder cancer	Dementia	Cerebrovascular disease	Liver disease	Suicide
LST daytime	0.002** (0.1341)			0.011* (0.1321)					
SUHI daytime	0.005** (0.1384)			0.015* (0.1232)					
UHS daytime				0.031* (0.2437)					
LST night-time	0.003** (0.0722)		0.002** (0.0612)						
SUHI night-time	0.004** (0.0725)		0.002** (0.0640)						
UHS night-time	0.004** (0.0872)		0.000*** (0.0533)	0.022* (0.0294)					
R^2^	0.171	0.183	0.252	0.280	0.292	5.291	4.340	5.314	4.415
F	2.31	2.41	3.77	4.45	4.53	0.32	0.23	0.21	0.10
Prob > chi^2^	0.0001	0.0001	0.0000	0.0034	0.0000	0.0001	0.0004	0.0000	0.0000

*R*^2^ Linear regression coefficient, *F F* statistic

Robust standard errors: *p < 0.05; **p < 0.01; ***p < 0.001

The basic unit of analysis in this section is the environmental variables and the excess risk of the different diseases investigated. The results of the statistical analysis report that the environmental variables show strong positive correlations with the excess risk of disease from stomach cancer, prostate cancer and lung cancer. These results, which statistically do not guarantee causality, are in line with the values obtained analytically and indicated above. In this way, both analytically and statistically, it is reported that the areas with the highest LST and/or SUHI also present a greater excess risk of cancer (stomach, lung and prostate) and vice versa. Therefore, a relationship between high temperatures and excess risk of disease could be intuited. The results of the Data Panel report a statistically significant and positive relationship of 99% between environmental variables and stomach and lung cancer. This relationship becomes 95% with prostate cancer. The values of *R*^2^, *F* statistic and Prob > chi^2^ obtained indicate a good concordance between the dependent and independent variables used, with an adjustment level greater than 99% significance since Prob > chi^2^ = 0.000.

## Discussion

This study analysed the space–time variability of the LST, SUHI and UHS during the year 2021 in the city of Granada, as well as its relationship with the variables NDVI, NDBI, PV and environmental pollution in order to assess how they may have influenced the risk of disease among the local population. A number of studies reports that changes in these indices and an increase in environmental contamination bear a significant impact on the increased development of diseases.

In the studied area, the highest values of the NDVI and PV indices are found in rural areas as opposed to urban areas, which present the lowest values. These variables are inversely related to the NDBI variable, in such a way that the highest values are found in urban areas as opposed to rural ones. The high rate of growth undergone by the city of Granada, largely owing to migration from rural zones in recent decades, has led to an increased NDBI. In contrast, there has been a decrease in rural areas with agricultural uses (green areas) since the nineteenth century in the Mediterranean basin (Benayas et al. 2007). The agricultural crisis that Spain suffered at the beginning of the twentieth century —involving economic development, high production costs, droughts, progressive industrialization and speculation, transforming green areas into urban ones to obtain high profits— are common circumstances explaining this type of coverage (Romero Díaz and Martínez Hernández 2014). Similar findings are reported by previous authors (Guha et al. 2018; Macarof and Statescu 2017; Shafizadeh-Moghadam et al. 2020) in other areas or cities, validating the results obtained here.

Regarding the city of Granada, the highest daytime LST and SUHI values occur in rural areas; at night, the highest LST and SUHI are seen for urban areas. It is evident, in turn, that the greatest increases in LST and SUHI occur in areas presenting lower NDVI and PV indices, which coincide with the areas that present higher NDBI values. Numerous studies confirm that during the early hours of the morning, solar radiation in areas of little vegetation or rural areas is greater than the radiation in urban areas. This is due to the shadows generated by buildings and trees that prevent the heating of enclosures and impermeable surfaces, and the cooling rates of vegetated areas (Li and Meng 2018; Yang et al. 2020a). Some studies carried out with satellite images show how vegetation has a cooling effect in urban areas (Lin et al. 2015; Tan et al. 2017; Yu et al. 2017), whereas warming occurs in areas having scarce vegetation and/or bare soils (Estoque et al. 2017; Lin et al. 2015). These effects are not only due to the processes of shading and evapotranspiration, but also depend on the rates of cooling or heating by convection and transpiration, which would alter the LST of an area and explain the behaviour of the SUHI, as confirmed in this investigation. Furthermore, the urban use of waterproof construction materials with high thermal absorption leads to a release of heat absorbed during the day, hence an increased LST at night. This means a greater variability of the LST in rural areas, producing an increase in the nocturnal SUHI phenomenon (Saaroni et al. 2018; Wu et al. 2019; Yang et al. 2020b). Such associations have been evidenced by statistical analysis giving strong positive correlations between the LST on the one hand and SUHI and NDBI on the other, and negative correlations with NDVI and PV indices. The reported findings are in line with the results of similar research efforts (Ahmed 2018; Dai et al. 2018; Guha et al. 2018; Luo and Wu 2021; Macarof and Statescu 2017; Shafizadeh-Moghadam et al. 2020; Sharma et al. 2021).

Our study evidences significant spatial variability of the areas classified as UHS between day and night readings. In the mornings, the ones classified as UHS are rural and outer urban areas. But at night, the UHS are located in urban areas. Again, this can be attributed to the variability of daytime and night-time LST and SUHI values. The strong positive correlations between these variables are likewise underlined by other studies (Guha et al. 2018; Shahfahad Talukdar et al. 2021; Sharma et al. 2021).

A very noteworthy relationship is detected between an excess risk of suffering any of the diseases investigated with the environmental variables studied (LST, SUHI and diurnal UHS), with the contamination variables (SO_2_ and NO_2_), and with the NDBI variable. No relationship could be established, however, with night-time values of LST, SUHI and UHS. It may therefore be affirmed that the inhabitants of Granada who live in areas entailing higher values of LST, SUHI and daytime UHS, NDBI, SO_2_ and NO_2_ run an excessive risk of contracting one of the diseases investigated. Statistical analysis shows that these variables are strongly related —areas characterized by higher temperatures have higher pollution rates and fewer green areas. Accordingly, citizens who live in areas identified as daytime UHS (and therefore with high daytime LST and SUHI temperatures) are at an increased risk, between 4 and 23%, of contracting the diseases of reference. As for the pollution variables, areas having the highest concentrations of pollution add an excess risk of 1 to 4% in conjunction with NO_2_, and between 1 and 8% for SO_2_. Finally, higher NDBI values could be tied to excess risk of disease anywhere between 1 and 18%. Numerous studies confirm that the incidence and severity of disease or health problems rises with increasing temperature (Heaviside et al. 2017; Shahmohamadi et al. 2011), population density (Tomlinson et al. 2011) and environmental pollution (Dominici et al. 2012; Pedersen et al. 2017; Rangel and Tomé 2022) validating the results obtained in this research.

Altogether, the evidence expounded here supports the fact that urban areas having higher daytime temperatures are of special importance when it comes to establishing mitigation measures to improve the quality of life by limiting the risk of disease.

## Conclusions

In recent decades, environmental study of urban areas in terms of environmental pollution and the alteration of urban climate has become a consolidated field of research. Its vital importance stems from our need to know what factors alter urban climates and what impact they have on the proliferation of diseases. These circumstances must be considered as fundamental in order to establish mitigation and correction measures for future urban proposals that improve the quality of life of citizens and allow minimizing the exacerbation of diseases with high mortality rates and that generate large economic costs for the citizens and government health systems.

The area studied here, during 2021, provides sound evidence that urban areas have comparatively low values of LST, SUHI and UHS during the day, while rural areas show higher values. During the night, however, urban areas have higher values of LST, SUHI and UHS. The rural ones have higher NDVI and PV indices; urban areas show higher values for the NDBI. Thus, certain relationships can be envisaged: for the diurnal environmental variables LST, SUHI and UHS; the environmental pollution variables NO_2_ and SO_2_ and the NDBI, in conjunction with an excess of risk in the development of the diseases specifically explored here. This finding underlines an urgent need to take measures to mitigate or minimize temperature, pollution and population density in order to ensure quality of life for local inhabitants and reduce the impact of diseases. To this end, we put forth strategies that have proven very effective in other urban areas: a promotion of green areas or free spaces with vegetation, the development of an efficient and sustainable public transport system, pedestrianization of urban areas and the use of low emissivity building materials. An increase in green areas will increase the plant cover that receives solar radiation but does not revert it to the atmosphere, as happens with impermeable materials and surfaces, thereby reducing both temperature and pollution.

In practice, our findings contribute to a comprehensive understanding of the interplay between daytime and night-time LST, SUHI, and UHS, NDVI, NDBI and PV indices and environmental contamination in the context of variability of excess risk of death/disease in the Mediterranean city of Granada. Urban planners and public administrations in charge of managing future growth areas might take note when making decisions on the most appropriate mitigation and resilience measures, as minimizing the variables that entail an excessive risk of disease is an endeavour of great importance for the entire population. It should not be forgotten that these diseases have high mortality rates among the population and a high economic cost for government health systems. The results of studies like ours could be extrapolated to other cities, given that the use of Sentinel 3 satellite images lies within the reach of the entire scientific community, and their use for determining LST and SUHI is straight forward.

### Limitations to the study

Regarding the limitations of the study carried out, it is considered necessary for future research to increase the number of years of analysis in order to corroborate a greater relationship between environmental variables and diseases. This research contemplates forty-eight satellite images but only from the year 2021. Therefore, increasing the number of years should be the next way of working. On the other hand, it is considered convenient to carry out new studies on other cities to verify if the variables outlined here are characteristics of this zone or climate or if they are repeated in other urban zones. This would allow the discovery of new variables or circumstances that could be related to the results of the investigation.

## Data Availability

Not applicable.
